# Deep sequencing of the TCRβ repertoire reveals T cell clonal expansion is associated with HTLV-1 proviral load in HAM/TSP patients

**DOI:** 10.1186/1742-4690-12-S1-O21

**Published:** 2015-08-28

**Authors:** Alessandra de Paula A Sousa, Kory R Johnson, Joan Ohayon, Jun Zhu, Paolo Muraro, Steven Jacobson

**Affiliations:** 1 Neuroimmunology Branch, Viral Immunology Section, National Institute of Neurological Disorders and Stroke, NIH, Bethesda, Maryland, USA; 2 Bioinformatics Section, National Institute of Neurological Disorders and Stroke, NIH, Bethesda, Maryland, USA; 3 Systems Biology Center, National Heart Lung and Blood Institute, Bethesda, Maryland, USA; 4 Division of Brain Sciences, Faculty of Medicine, Imperial College London, UK

## 

HTLV-1 associated myelopathy/tropical spastic paraparesis (HAM/TSP) is associated with a chronic inflammatory central nervous system in which the host immune response against HTLV-1 infection is considered immunopathogenic. We have applied a new unbiased deep sequencing method of TCR repertoire to accurately measure the diversity and degree of clonal expansion of peripheral circulating T-cells in patients with HAM/TSP compared to age, gender, and ethnicity matched healthy controls. TCR libraries were generated using 5'rapid amplification of cDNA ends to amplify the V-D-J genes of unsorted T-cells obtained from peripheral blood. Over 100 million reads were analyzed using MiGec software (Shugay, et al., Nat Methods. 2014 Jun;11(6):653-5), that align and match the human TCR β-chain nucleotide sequences through IMGT database. T-cell clonotypes with high-quality CDR3 sequence were selected by a molecular identifier PCR strategy, which allowed for filtering PCR errors and correct for artificial sequences generated on a HiSeq 2000 Illumina system platform. By calculating the coefficient of variation of our dataset we considered clonal expansion as those unique TCR clonotypes showing reads >8. While we did not observe any significant statistical differences of the number of single clones (clonotypes with 1 read, P=0.62) between HAM/TSP patients and healthy controls groups, by contrast, a higher clonal expansion of the T-cell repertoire was observed in HAM/TSP patients when compared to healthy controls (unpaired parametric T-test with P=0.026). Interestingly, this clonal expansion of the HAM/TSP TCR repertoire correlated with the HTLV-I provirus load (as defined by digital PCR quantification of the HTLV-I tax gene) (r=0.64, P=0.008). Our findings strongly suggest that in this chronic neurological disorder, a strong dysregulated T-cell response may be driven by HTLV-I and that strategies to decrease virus may be of clinical benefit.

